# Risk factors of postoperative major adverse cardiac events after radical cystectomy: implication of diastolic dysfunction

**DOI:** 10.1038/s41598-019-50582-6

**Published:** 2019-10-01

**Authors:** In-Jung Jun, Junghwa Kim, Hyun-Gyu Kim, Gi-Ho Koh, Jai-Hyun Hwang, Young-Kug Kim

**Affiliations:** 1 Department of Anesthesiology and Pain Medicine, Kangnam Sacred Heart Hospital, University of Hallym College of Medicine, Seoul, Korea; 2 Department of Anesthesiology and Pain Medicine, Asan Medical Center, University of Ulsan College of Medicine, Seoul, Korea

**Keywords:** Risk factors, Risk factors, Bladder, Bladder

## Abstract

Radical cystectomy, which is a standard treatment of muscle invasive and high-grade non-invasive bladder tumour, is accompanied with high rates of postoperative complications including major adverse cardiac events (MACE). Diastolic dysfunction is associated with postoperative complications. We evaluated perioperative risk factors including diastolic dysfunction related with MACE within 6 months after radical cystectomy. The 546 patients who underwent elective radical cystectomy were included. Diastolic dysfunction was defined as early transmitral flow velocity (E)/early diastolic mitral annulus velocity (e′) > 15. Logistic regression analysis, Kaplan-Meier survival analysis and log-rank test were performed. MACE within 6 months after radical cystectomy developed in 43 (7.9%) patients. MACE was related with female (odds ratio 2.546, 95% confidence interval 1.166–5.557, P = 0.019) and diastolic dysfunction (odds ratio 3.077, 95% confidence interval 1.147–8.252, P = 0.026). The 6-month mortality were significantly higher in the MACE group, and hospital stay and intensive care unit stay were significantly longer in the MACE group compared to the non-MACE group. Accordingly, preoperative diastolic dysfunction (E/e′ > 15) was related with postoperative MACE and MACE was related with 6-month survival after radical cystectomy. These results suggest that preoperative diastolic dysfunction can provide useful information on postoperative complications.

## Introduction

Radical cystectomy is regarded as a standard treatment in patients with muscle invasive and high-grade non-invasive bladder tumour^1,2^. Radical cystectomy is classified as a major operative procedure and is accompanied with high postoperative complication rates among urological operations. High rates of morbidity after radical cystectomy is due to not only its technical challenges of the operation but also the characteristics of the patients with mostly elderly population with associated underlying comorbidities^3^. Even with considerable advances in surgical and anaesthetic techniques, early complication rates occurring within 90 days after radical cystectomy still ranges over 58–64%^4–6^. Among early complications after radical cystectomy, cardiac complications are reported at the range of 1.4–4.1% and significantly affect postoperative morbidity and mortality^7^.

Diastolic dysfunction presents diminished left ventricular compliance due to abnormal left ventricular relaxation and filling during diastole, and subsequently increased left ventricular filling pressure. Sustained increase in left ventricular filling pressure can be intolerable in volume adjustment and hence is associated with perioperative cardiac events^8^. In this regard, diastolic dysfunction may demonstrate on primary cardiac events^9^. The clinical implication of diastolic dysfunction may be particularly relevant in surgeries including mostly elderly patients such as radical cystectomy. However, the prognostic role of diastolic dysfunction as a preoperative risk factor of postoperative major adverse cardiac events (MACE) in patients undergoing radical cystectomy has not been clearly explained.

Therefore, in the present study, we aimed to evaluate independent risk factors including diastolic dysfunction related with MACE within 6 months following radical cystectomy in bladder cancer patients. In addition, postoperative outcomes such as acute kidney injury, pulmonary complications, 6-month mortality, and lengths of intensive care unit stay and hospital stay were compared between MACE group (patients who developed MACE) and non-MACE group (those who did not).

## Results

**Figure 1:**
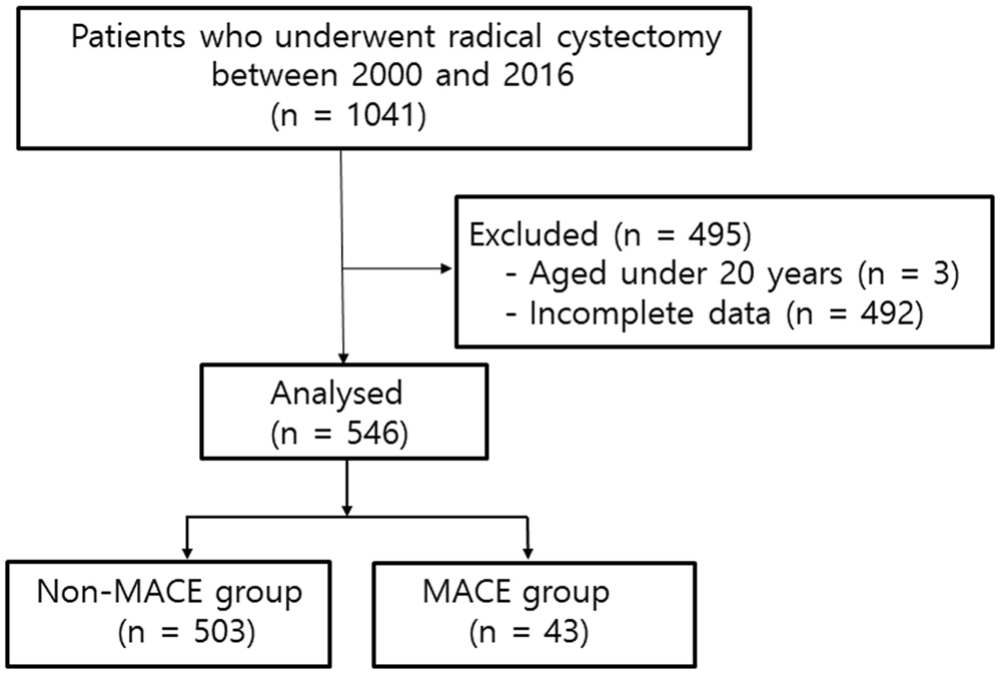
Study flow diagram. MACE = major adverse cardiac events.

We analysed 546 patients who underwent radical cystectomy during the study period (Fig. 1). We excluded 492 patients who did not undergo preoperative echocardiography and classified them as incomplete data. Of the 546 patients, 503 patients were included in non-MACE group and 43 patients were included in MACE group. Of 43 MACE group patients, 2 had myocardial infarction, 21 had arrhythmia, 12 had heart failure, 9 had cerebrovascular accident, and 8 had nonfatal cardiac arrest in the postoperative period.

Patient characteristics, medications, clinical characteristics, laboratory data, and transthoracic echocardiography parameters of 546 patients are described in Table 1. The MACE and the non-MACE groups showed significant differences in terms of age, gender, American Society of Anesthesiologist (ASA) physical status, diuretic, aspirin, vasodilator, early transmitral filling velocity (E)/early diastolic septal mitral annulus velocity (e′), and E/e′ > 15 (Table 1). The two groups did not show significant differences in their intraoperative data except urinary diversion type (Table 2).

**Table 1:** Preoperative data of 546 patients who underwent radical cystectomy.

	Non-MACE group (n = 503)	MACE group (n = 43)	P value
Age (yrs)	64.1 ± 9.7	67.7 ± 10.3	0.019
Gender			0.011
Male	438 (87.1)	31 (72.1)	
Female	65 (12.9)	12 (27.9)	
Body mass index (kg/m^2^)	24.3 ± 3.2	24.3 ± 3.1	0.989
ASA physical status			<0.001
Class 1	37 (7.4)	0 (0)	
Class 2	432 (85.9)	33 (76.7)	
Class 3	34 (6.8)	10 (23.3)	
Diabetes mellitus	104 (20.7)	11 (25.6)	0.449
Hypertension	222 (44.1)	21 (48.8)	0.552
Hyperlipidaemia	15 (3.0)	3 (7.0)	0.159
Heart failure	5 (1.0)	1 (2.3)	0.421
Coronary artery disease	18 (3.6)	3 (7.0)	0.224
Cerebrovascular disease	17 (3.4)	3 (7.0)	0.203
Preoperative medications			
ACEI	54 (10.7)	6 (14.0)	0.517
Beta-blocker	16 (3.2)	2 (4.7)	0.645
Calcium-channel blocker	139 (27.6)	16 (37.2)	0.181
Diuretic	21 (4.2)	5 (11.6)	0.028
Aspirin	27 (5.4)	6 (14.0)	0.023
Plavix	11 (2.2)	2 (4.7)	0.273
Vasodilator	35 (7.0)	7 (16.3)	0.028
Tumour stage			0.383
1	15 (3.0)	3 (7.0)	
2	313 (62.4)	29 (67.4)	
3	115 (22.9)	7 (16.3)	
4	59 (11.8)	4 (9.3)	
Tumour grade			0.725
2	30 (6.0)	2 (4.7)	
3	473 (94.0)	41 (95.3)	
Neo-adjuvant chemotherapy	90 (17.9)	8 (18.6)	0.907
Adjuvant chemotherapy	227 (45.1)	15 (34.9)	0.194
Preoperative laboratory values			
Haematocrit (%)	37.0 ± 5.3	36.8 ± 5.2	0.875
Creatinine (mg/dL)	1.0 ± 0.5	1.2 ± 1.2	0.336
C-reactive protein (mg/dL)	1.0 ± 2.4	1.2 ± 2.5	0.703
High density lipoprotein (mg/dL)	46.2 ± 11.5	44.4 ± 12.9	0.676
Low density lipoprotein (mg/dL)	111.0 ± 30.6	106.2 ± 38.7	0.720
Albumin (g/dL)	3.7 ± 0.5	3.7 ± 0.6	0.481
Uric acid (mg/dL)	5.3 ± 1.5	5.6 ± 1.5	0.138
Preoperative TTE findings			
Ejection fraction (%)	62.0 ± 4.6	60.8 ± 4.7	0.080
E/e′	10.1 ± 2.9	12.2 ± 4.7	0.006
E/e′ > 15	21 (4.2)	7 (16.3)	0.004
E/A	0.87 ± 0.3	0.80 ± 0.3	0.856
Deceleration time (msec)	217 ± 49.1	203 ± 51.0	0.990
Left ventricular end-diastolic volume (mL)	91.5 ± 26.0	93.7 ± 31.7	0.073
Study period			0.284
1	181 (36.0)	19 (44.2)	
2	322 (64.0)	24 (55.8)	

Data are expressed as mean ± standard deviation or number of patients (%). MACE = major adverse cardiac events; ASA = American Society of Anesthesiologist; ACEI = angiotensin-converting enzyme inhibitor; TTE = transthoracic echocardiography; E/e′ = early transmitral filling velocity/early diastolic septal mitral annulus velocity, E/A = early transmitral filling velocity/late transmitral filling velocity. Years 2005 to 2010 were classified as period 1 and years 2011 to 2016 were classified as period 2.

**Table 2:** Intraoperative data of 546 patients who underwent radical cystectomy.

	Non-MACE group (n = 503)	MACE group (n = 43)	P value
Anaesthesia time (min)	447.8 ± 97.9	465.5 ± 96.7	0.256
Operation time (min)	410.0 ± 96.0	419.9 ± 98.7	0.521
Urinary diversion type			0.034
Ileal conduit	185 (36.8)	23 (53.5)	
Ileal neobladder	318 (63.2)	20 (46.5)	
Crystalloid administered (mL)	3412.5 ± 1237.1	3373.3 ± 1539.2	0.845
Colloid administered (mL)	565.8 ± 444.6	643.0 ± 375.7	0.269
Red blood cell transfusion	300 (59.6)	30 (69.8)	0.192
Use of vasopressor/inotropic	181 (36.0)	18 (41.9)	0.442

Data are expressed as mean ± standard deviation or number of patients (%). MACE = major adverse cardiac events.

Univariate analysis showed that age, gender, diuretic, aspirin, vasodilator, E/e′ > 15, and urinary diversion type were associated with MACE within 6 months after radical cystectomy. After multivariate analysis, female (odds ratio = 2.546, 95% confidence interval = 1.166–5.557, P = 0.019) and E/e′ > 15 (odds ratio = 3.077, 95% confidence interval = 1.147–8.252, P = 0.026) were related with MACE within 6 months after radical cystectomy (Table 3).

**Table 3:** Univariate and multivariate regression analyses to identify factors that associate with MACE within 6 months after radical cystectomy.

	Univariate analysis		Multivariate analysis	
	Odds ratio (95% CI)	P value	Odds ratio (95% CI)	P value
Age	1.044 (1.007–1.083)	0.020	1.028 (0.987–1.071)	0.180
Gender				
Male	1.000		1.000	
Female	2.608 (1.275–5.335)	0.009	2.546 (1.166–5.557)	0.019
Body mass index	0.999 (0.907–1.101)	0.989		
ASA physical status				
Class 1	1.000			
Class 2	0.480 (0.175–1.316)	0.154		
Class 3	2.176 (0.675–7.014)	0.193		
Diabetes mellitus	1.319 (0.643–2.705)	0.450		
Hypertension	1.208 (0.648–2.253)	0.552		
Hyperlipidaemia	2.440 (0.678–8.783)	0.172		
Heart failure	2.371 (0.271–20.770)	0.435		
Coronary artery disease	2.021 (0.571–7.153)	0.275		
Cerebrovascular disease	2.144 (0.603–7.627)	0.239		
ACEI	1.348 (0.544–3.342)	0.519		
Beta-blocker	1.485 (0.330–6.682)	0.607		
Calcium-channel blocker	1.552 (0.811–2.968)	0.184		
Diuretic	3.020 (1.079–8.457)	0.035	1.650 (0.477–5.710)	0.429
Aspirin	2.859 (1.110–7.361)	0.029	1.780 (0.561–5.648)	0.328
Plavix	2.182 (0.468–10.177)	0.321		
Vasodilator	2.600 (1.079–6.265)	0.033	1.360 (0.428–4.315)	0.602
Tumour stage				
1	1.000			
2	0.463 (0.127–1.694)	0.245		
3	0.304 (0.071–1.305)	0.109		
4	0.339 (0.068–1.680)	0.185		
Tumour grade				
2	1.000			
3	1.300 (0.300–5.635)	0.726		
Neo-adjuvant chemotherapy	1.049 (0.471–2.337)	0.907		
Adjuvant chemotherapy	0.651 (0.340–1.249)	0.197		
Haematocrit	0.995 (0.939–1.055)	0.874		
Creatinine	1.304 (0.946–1.798)	0.105		
C-reactive protein	1.026 (0.900–1.170)	0.703		
High density lipoprotein	0.986 (0.924–1.053)	0.672		
Low density lipoprotein	0.995 (0.967–1.023)	0.716		
Albumin	0.794 (0.418–1.507)	0.480		
Uric acid	1.166 (0.952–1.430)	0.138		
Ejection fraction	0.946 (0.889–1.007)	0.081		
E/e′ > 15	4.463 (1.779–11.199)	0.001	3.077 (1.147–8.252)	0.026
E/A	0.430 (0.124–1.491)	0.183		
Deceleration time	0.993 (0.986–1.001)	0.070		
Left ventricular end-diastolic volume	1.003 (0.992–1.015)	0.603		
Study period				
1	1.000			
2	0.710 (0.379–1.332)	0.286		
Operation time	1.001 (0.998–1.004)	0.520		
Urinary diversion type				
Ileal conduit	1.000			
Ileal neobladder	0.506 (0.270–0.946)	0.033	0.950 (0.447–2.018)	0.893
Crystalloid	1.000 (1.000–1.000)	0.845		
Colloid	1.000 (1.000–1.001)	0.269		
Red blood cell transfusion	1.562 (0.795–3.066)	0.195		
Use of vasopressor/inotropic	1.281 (0.680–2.411)	0.443		

MACE = major adverse cardiac events; CI = confidence interval; ASA = American Society of Anesthesiologist; ACEI = angiotensin-converting enzyme inhibitor; E/e′ = early transmitral filling velocity/early diastolic septal mitral annulus velocity, E/A = early transmitral filling velocity/late transmitral filling velocity. Years 2005 to 2010 were classified as period 1 and years 2011 to 2016 were classified as period 2.

**Figure 2:**
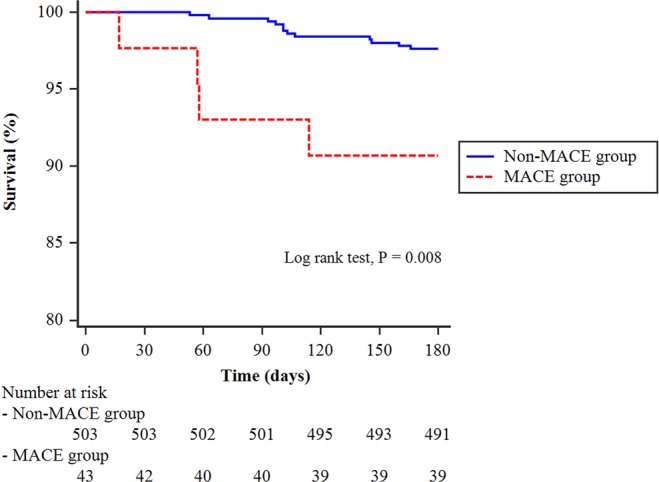
Kaplan-Meier curves of 6-month survival in the non-MACE group (blue) and the MACE group (red) of patients who underwent radical cystectomy. MACE = major adverse cardiac events.

Compared with the non-MACE group, the MACE group had significantly higher prevalence of acute kidney injury, pulmonary complications during hospitalization after surgery, and 6-month mortality (Table 4). Hospital stay and intensive care unit stay were significantly longer in the MACE group compared to the non-MACE group. The Kaplan-Meier curve showed that 6-month survival rate was significantly higher in non-MACE group than in MACE group (P = 0.008) (Fig. 2).

**Table 4:** Postoperative outcomes of 546 patients who underwent radical cystectomy.

	Non-MACE group (n = 503)	MACE group (n = 43)	P value
Acute kidney injury	17 (3.4)	6 (14.0)	0.001
Pulmonary complications	31 (6.2)	9 (20.9)	<0.001
6-month mortality	12 (2.4)	4 (9.3)	0.030
Intensive care unit stay (day)	4.7 ± 16.8	17.2 ± 31.2	0.013
Hospital stay (day)	28.7 ± 20.1	45.6 ± 65.6	<0.001

Data are expressed as mean ± standard deviation or number of patients (%). MACE = major adverse cardiac events.

## Discussion

The major findings in this study showed that preoperative diastolic dysfunction (E/e′ > 15) and female gender were related with MACE within 6 months after radical cystectomy in bladder cancer patients. Furthermore, postoperative MACE was related with 6-month survival after radical cystectomy.

The present study revealed that preoperative diastolic dysfunction was related with postoperative MACE after radical cystectomy. It is well acknowledged that a history of congestive heart failure is associated with higher risk of postoperative morbidity as well as mortality following non-cardiac surgery^10^. Although congestive heart failure includes both systolic and diastolic heart failure, clinical research has been relatively focused on systolic heart failure, and diastolic heart failure has not been thoroughly studied due to the lack of a universal, non-invasive classification method and meticulous approach in diagnosis^11,12^. Also, the correlation of preoperative diastolic dysfunction and postoperative MACE has not been well described after non-cardiac surgery. Significant portion of elderly patients undergoing cardiac or non-cardiac surgeries commonly show diastolic dysfunction with normal left ventricular ejection fraction, as ranging over 50% as reported by Phillip *et al*.^13^. Thus, the clinical implication of diastolic dysfunction may be particularly important in a type of surgery with extensive long operation duration with mostly elderly patients. Previous studies have evaluated diastolic dysfunction and MACE after non-cardiac surgery^14–16^. However, the present study has strength that we evaluated the relationship of diastolic dysfunction and postoperative MACE in a large number of homogeneous elderly patients undergoing high-risk non-cardiac surgery, which is radical cystectomy.

Preoperative diastolic dysfunction is reported to be associated with postoperative cardiac complications, particularly in the elderly and there may be following reasons^15^. First, diastolic dysfunction can induce the increase in left ventricular filling pressure and the intolerance in volume adjustment, and subsequently poor cardiac outcomes^17^. Second, diastolic dysfunction may impair coronary flow^18^. Galderisi *et al*. have found that coronary flow reserve was reduced in subjects with diastolic dysfunction^19^. It was noted that increases in left ventricular wall stress in individuals with normal physiology increases the coronary flow reserve and subsequently increases myocardial blood flow^20^. However, this is often impaired in individuals under pathologic states such as left ventricular diastolic dysfunction, advanced age with possible cardiac structural abnormalities, or subclinical myocardial fibrosis^20–22^. In addition, tachycardia may aggravate coronary flow reduction in individuals with reduced coronary flow reserve^18^. Thus, our group of patients with increased E/e′ undergoing radical cystectomy, which need a large amount of intraoperative fluid administration, may be prone to coronary flow impairment followed with subendocardial ischaemia and hence have resulted in postoperative MACE. Third, inevitable volume depleting condition as well as volume loading condition from long operation time with substantial amount of blood loss may affect haemodynamic instability and left ventricular remodelling which may have also contributed to postoperative MACE after radical cystectomy.

Systolic heart failure is considered to be associated with MACE in several previous reports. Ewe *et al*. have reported significantly high rates of MACE in patients with baseline left ventricular ejection fraction <50% compared to normal left ventricular ejection fraction in patients undergoing transcatheter aortic valve implantation^23^. Also, left ventricular ejection fraction <50% was an independent predictor of MACE in elderly patients undergoing non-cardiac surgery^24^. However, most of the patients in our study had normal range of ejection fraction without evidence of heart failure. Therefore, in the present study, systolic heart failure was not found as a risk factor of MACE after radical cystectomy.

We found that female gender was also related with MACE within 6 months after radical cystectomy. There has been conventional view that sex steroid hormones exhibit different actions against cardiac stress, although the details on the mechanism of action are unresolved^25^. The incidence of cardiovascular events increases in post-menopause women; therefore, elderly women experience higher rate of cardiovascular events and mortality compared to age-matched men^25^. Considering that the patients in the present study had a mean age of 64 years, sex steroid hormone may have influenced the manifestation of MACE after radical cystectomy in the present study. In addition, several studies have reported gender-specific discrepancy after radical cystectomy, with female gender having poor postoperative outcomes compared to male gender^26,27^. It has been suggested that different pelvic anatomy renders the gender difference in disease progression and postoperative outcomes. Prostatic capsule and prostatic urethra may block the angio-lymphatic extension of cancer cell in male gender^28^. Also, since female bladder neck muscle is not as sturdy as male, lymphatic drainage pattern may have differed and hence influenced the gender-specific discrepancy.

Here, we have shown that post-operative MACE was significantly related with post-operative mortality. In line with the present study, Beattie *et al*. noted that postoperative MACE increased risk of mortality in patients undergoing non-cardiac surgery^29^. The authors suggested that perioperative myocardial injury cannot overcome the surgical stress, and inability to cope with the stress may have turned out as high mortality^29^. Furthermore, clinically silent troponin elevation from myocardial injury during postoperative period is known to increase the long-term mortality after major non-cardiac surgery^30^.

The present study has limitations stemming from its retrospective observational design, which is prone to selection bias. Moreover, as we only included patients who underwent preoperative transthoracic echocardiography, our findings need to be interpreted with caution prior to wider generalisation. Second, our results from the logistic regression analysis may have been limited by the relatively small number of patients with MACE. Third, the Doppler tissue imaging parameters to evaluate the diastolic dysfunction were limited. As for E/e′, a single measurement of septal mitral annular velocity was only attainable. However, it has been reported that there are no significant differences among E/e′ of septal mitral annular velocity, E/e′ of lateral mitral annular velocity, and the average of E/e′ in their predictive value for cardiac events^31^. Also, there was no additional predictive value in the intra-individual variation of E/e′^31,32^. We therefore assumed that a single measurement of septal E/e′ can be used as a parameter of left ventricular filling pressure and hence be a predictive parameter of MACE after radical cystectomy.

In conclusion, this study showed that preoperative diastolic dysfunction, which was evaluated by E/e′ > 15, was related with MACE within 6 months after radical cystectomy in patients who underwent preoperative transthoracic echocardiography. Postoperative MACE was related with 6-month survival after radical cystectomy. These results suggest that preoperative evaluation of left ventricular diastolic function with E/e′ may be helpful in predicting cardiac outcomes after radical cystectomy.

## Methods

### Study design and participants

The protocols of this study were approved by the Institutional Review Board of Asan Medical Center (approval number: 2017-0728). We included patients who underwent elective radical cystectomy at our institution between November 2000 and December 2016. The demographic and clinical characteristics, laboratory data, and intraoperative and postoperative data were collected from digitized patient records (Asan Medical Center Information System Electronic Medical Record) and the database at Asan Medical Center. We excluded patients who were below 20 years of age and had missing data. The requirement for written informed consents was waived by the Institutional Review Board due to retrospective study design. All methods were carried out in accordance with relevant guidelines and regulations.

### Anaesthetic and surgical techniques

General anaesthesia was induced with either thiopental or propofol intravenous injection with rocuronium. Anaesthesia was maintained using volatile anaesthetic agents (sevoflurane, isoflurane, or desflurane) with 50% oxygen/air mixture. Conventional parameters including electrocardiography, heart rate, peripheral oxygen saturation, and continuous arterial blood pressure were used for haemodynamic and fluid management. Core temperature was monitored by using oesophageal stethoscope.

Crystalloid solutions (plasma solution A (CJ Pharmaceutical, Seoul, Korea) or lactated Ringer’s solution) and colloid solutions (Volulyte (Fresenius Kabi, Bad Homburg, Germany) or 5% albumin) were administered. Crystalloid solution was administered at a rate of 6–10 ml/kg/hr, and colloid solution was used at anaesthesiologist’s discretion. Packed red blood cells were transfused to maintain a haemoglobin concentration greater than 8 g/dL.

Surgical technique was performed as follows^33,34^. Pelvic lymphadenectomy and radical cystectomy were carried out according to the standard technique used at our centre. Standard lymph node dissection including the external iliac, distal common iliac, obturator, hypogastric, and perivesical lymph nodes or extended lymph node dissection to the extent of the distal aorta, proximal common iliac artery, and vena cava was done as determined by the urologic surgeon. Subsequent urinary diversion of ileal neobladder or ileal conduit was carried out after determination of the type by the urologic surgeon.

### Measurements and definitions of variables

The variables included patient characteristics (age, gender, ASA physical status, body mass index, and comorbidities including diabetes mellitus, hypertension, hyperlipidaemia, heart failure, coronary artery disease, and cerebrovascular disease), preoperative medications (beta-blocker, angiotensin-converting enzyme inhibitor, diuretic, calcium-channel blocker, aspirin, plavix, and vasodilator), clinical characteristics (tumour stage, tumour grade, neo-adjuvant chemotherapy, and adjuvant chemotherapy), preoperative laboratory values (serum haematocrit, creatinine, high density lipoprotein, low density lipoprotein, C-reactive protein, albumin, and uric acid), preoperative transthoracic echocardiography parameters (ejection fraction, E/e′, early transmitral filling velocity (E)/late transmitral filling velocity (A), deceleration time, and left ventricular end-diastolic volume), intraoperative data (anaesthesia time, operation time, urinary diversion type, administered amounts of crystalloid and colloid solutions, red blood cell transfusion, and use of vasopressor/inotropic), and postoperative data (MACE, acute kidney injury, pulmonary complications, mortality, and lengths of intensive care unit stay and hospital stay). The study period was divided into the past half period (period 1) and the recent half period (period 2) by the average of the period; Years 2005 to 2010 were classified as period 1 and years 2011 to 2016 were classified as period 2.

Diabetes mellitus was defined as a history of uncontrolled blood glucose level with preoperative history of antihyperglycaemic medication. Hypertension was defined as systolic arterial blood pressure >140 mmHg and diastolic blood pressure >90 mmHg with preoperative history of antihypertensive medication. Hyperlipidaemia was defined as elevated serum lipoproteins or preoperative history of antihyperlipidemic medication. Heart failure was defined as left ventricular systolic dysfunction with ejection fraction less than 40%. Cerebrovascular disease was defined as a history of stroke, cerebral haemorrhage, or carotid artery stent or angioplasty. Coronary artery disease was defined as previous diagnosis of ischaemic heart disease by a cardiologist. Preoperative diuretic included thiazide, furosemide, and spironolactone. Preoperative vasodilator included isoket, nitroglycerine, and nitrate.

Tumour stage was assessed by the 2010 American Joint Committee on Cancer tumour-node-metastasis staging system^35^. Tumour grade was assessed by the 2016 World Health Organization grading system^36^. A portion of patients received neo-adjuvant or adjuvant chemotherapy according to the decision of surgeons and oncologists. Neo-adjuvant chemotherapy comprised the combinations of gemcitabine/cisplatin, methotrexate/vinblastine sulfate/cisplatin/doxorubicin, or gemcitabine/carboplatin. Adjuvant chemotherapy comprised the combinations of gemcitabine/cisplatin, methotrexate/vinblastine sulfate/cisplatin/doxorubicin, or cyclophosphamide/cisplatin.

Preoperative transthoracic echocardiography was performed 2 months prior to surgery by experienced sonographers. Preoperative echocardiography was performed as determined by the attending anaesthesiologists and urologists. The parameters included left ventricular ejection fraction, E/e′, E/A, deceleration time, and left ventricular end-diastolic volume. The E and A were measured from the apical four-chamber view, and e′ was measured by pulsed wave Doppler with a sample volume from the septal site of the mitral annulus. Then, the E/e′ was calculated and used to determine increased left ventricular filling pressure^37^. Among various indices of indicating diastolic dysfunction, E/e′ is most widely used as a parameter of indicating left ventricular filling pressure^16,38^. We used E/e′ > 15 as the optimal cut off value since this best predicts increased left ventricular end-diastolic pressure^37,39^. Hypotensive episode was defined as mean arterial blood pressure below threshold of 60 mmHg with the duration of more than 5 minutes^40^. Vasopressor/inotropic such as phenylephrine, ephedrine, or norepinephrine was used when the hypotensive episode occurred.

Postoperative MACE was defined based on the European Perioperative Clinical Outcome definitions^41^. Postoperative MACE was recorded if the patient had one of the following complications within 6 months after radical cystectomy; acute myocardial infarction, arrhythmia, heart failure, cerebrovascular accident, or nonfatal cardiac arrest. Acute myocardial infarction was defined as increase in troponin, serum cardiac marker with one of the ischaemic symptoms; newly significant ST segment or T wave changes on electrocardiogram, development of pathological Q wave on electrocardiogram, or echocardiographic evidence of new regional wall motion abnormality. Arrhythmia included atrial fibrillation, atrial flutter, or second- or third-degree atrioventricular conduction block. Heart failure included newly developed postoperative left ventricular systolic dysfunction with ejection fraction less than 40%. Cerebrovascular accident included transient ischaemic attack, stroke, or cerebral haemorrhagic events during postoperative period. Non-fatal cardiac arrest was defined as the absence of cardiac rhythm or chaotic rhythm requiring cardiac life support.

Acute kidney injury was defined by Kidney Disease Improving Global Outcomes (KDIGO) guidelines^41^. Postoperative acute kidney injury was defined as an increase in serum creatinine by ≥0.3 mg/dL within 2 days after surgery or an increase in serum creatinine to ≥1.5 times baseline within 7 days after surgery^41^. Postoperative pulmonary complications were defined as newly diagnosed acute lung injury or newly diagnosed respiratory infection with one or more of the following criteria: lung opacity, fever, new sputum, white blood cell count >12 × 10^3^/μL, or newly detected pleural effusion demonstrated on chest radiograph during hospitalization after surgery^41^. Postoperative 6-month mortality was defined as all-cause mortality within 6 months after radical cystectomy.

### Statistical analysis

Continuous variables are expressed as mean ± standard deviation, and categorical variables are expressed as number (percentage). For comparison of demographics and intraoperative and postoperative characteristics between the MACE group and non-MACE group, chi-square test or the Fisher’s exact test was used for categorical variables and Mann-Whitney U test or the Student’s t-test was used for continuous variables.

Univariate and multivariate logistic regression analyses were conducted to identify risk factors that were associated with MACE within 6 months after radical cystectomy. The most relevant factors associated with postoperative MACE were independently assessed in univariate logistic regression analysis and the variables with a P < 0.05 were assessed in the multivariable logistic regression analysis.

The Kaplan-Meier method was used to describe the distribution of survival time within 6 months after radical cystectomy. The log-rank test was performed to compare differences in survival rates of the MACE group and non-MACE group. All reported *P* values were 2-sided, and those <0.05 were considered statistically significant. SPSS Version 23.0 (IBM Corp., Armonk, NY, USA) was used for all data manipulations and statistical analyses.

## Data Availability

The data used in the present study are available from the corresponding author upon reasonable request.
